# Population-specific transcriptomic shifts underlie secondary metabolic diversification in *Aspergillus flavus* Link and the domestication of *Aspergillus oryzae* (Ahlb.)

**DOI:** 10.3897/imafungus.17.195218

**Published:** 2026-10-07

**Authors:** Milton T. Drott, Yen-Wen Wang, E. Anne Hatmaker, Nayanna M. Mercado-Soto, J. Mitch Elmore, Dianiris Luciano-Rosaro, Jae-Hyuk Yu, Antonis Rokas, John G. Gibbons, Anna Huttenlocher, Kunlong Yang, Nancy P. Keller

**Affiliations:** 1 U.S. Department of Agriculture-Agricultural Research Service, Cereal Disease Laboratory, St. Paul, MN 55108, USA U.S. Department of Agriculture-Agricultural Research Service, Cereal Disease Laboratory St. Paul United States of America; 2 Department of Biostatistics, Yale School of Public Health, New Haven, CT, USA Department of Biostatistics, Yale School of Public Health New Haven United States of America; 3 Department of Plant Pathology, University of Georgia, Athens, GA, USA Department of Plant Pathology, University of Georgia Athens United States of America; 4 Department of Medical Microbiology and Immunology, University of Wisconsin-Madison, Madison, WI, USA Department of Medical Microbiology and Immunology, University of Wisconsin-Madison Madison United States of America; 5 U.S. Department of Agriculture-Agricultural Research Service, Food Quality Laboratory, Beltsville, MD, USA U.S. Department of Agriculture-Agricultural Research Service, Food Quality Laboratory Beltsville United States of America; 6 Department of Bacteriology, University of Wisconsin, Madison, WI, USA Department of Bacteriology, University of Wisconsin Madison United States of America; 7 Department of Biological Sciences, Vanderbilt University, Nashville, TN, USA Department of Biological Sciences, Vanderbilt University Nashville United States of America; 8 Department of Food Science, College of Natural Sciences, University of Massachusetts Amherst, Amherst, Massachusetts, USA Department of Food Science, College of Natural Sciences, University of Massachusetts Amherst Amherst United States of America; 9 Department of Plant Pathology, University of Wisconsin, Madison, WI, USA Department of Plant Pathology, University of Wisconsin Madison United States of America

**Keywords:** Evolution of secondary metabolism, mycotoxins, population transcriptomics

## Abstract

The genomic signatures of microbial domestication remain poorly understood within the context of natural population variation. Here, we demonstrate that *Aspergillus oryzae*, the filamentous fungus used in soy sauce and sake production, shares more recent ancestry with a predominantly northern, largely non-aflatoxigenic population of *Aspergillus flavus* (population C). *A. oryzae* isolates also overlap with the recently described, clinically enriched *A. flavus* population D, suggesting multiple domestication events. All isolates tested retained pathogenicity in a zebrafish infection model. At the transcriptomic level, populations are significantly differentiated and respond differently to spore density, suggesting population-specific adaptations to ecological conditions. Although most transcriptomic variation was consistent with phylogenetic relationships, several co-expression modules exhibited significantly greater divergence than expected from phylogeny alone, suggesting that phenotypic diversification may sometimes occur through the reorganization of transcriptomic architectures. *A. oryzae* also displayed elevated expression of a carbohydrate-metabolism module; however, this pattern was consistent with phylogenetic differentiation, suggesting that enhanced carbohydrate metabolism may have begun evolving prior to domestication or may be most apparent under fermentation-relevant conditions. Population-specific variation is also evident among secondary metabolite (SM) gene clusters. While *A. oryzae* shows markedly reduced expression of aflatoxin biosynthesis genes, this trend does not extend across the entire secondary metabolome. Using machine-learning-based gene regulatory network inference, we identified population-specific transcriptomic differences linked to distinct transcription factors, with evidence for both cis- and trans-acting regulatory divergence, but no changes in global regulators such as *laeA*. Together, these findings provide new insights into the domestication of *A. oryzae*, its global significance, and the microevolution of fungal secondary metabolic pathways.

## Introduction

The domestication of plants and animals is often cited as a key factor in explaining the success of burgeoning human civilizations. The impact of domestication on specific animal traits has been studied since Darwin (Darwin 1868), leading to the definition of ‘domestication syndromes,’ that define similar patterns of co-occurring traits across domesticated organisms. Similar patterns have also been described among domesticated plants (e.g., barley, diploid einkorn wheat, and tetraploid emmer wheat [Brown et al. 2009b]). While the phenotypes of domestication have been well described, disentangling the evolutionary histories of these organisms has often required insights from genomics. Recent pangenomic studies have demonstrated that dogs in the Near East and in Africa are descended from a specific population of Eurasian wolves, suggesting admixture or an independent domestication event (Bergström et al. 2022). Clarifying the ancestry of domesticated populations is essential for identifying how differences in gene content or genetic differentiation may contribute to phenotypic differentiation (Gong et al. 2023). Despite progress in understanding the evolution of charismatic or economically important domesticated plants and animals, microbial domestication has been relatively overlooked (Steensels et al. 2019). Domesticated microbes, like plants and animals, have shaped human history and the way we eat today. These organisms, however, do not always exhibit the fundamental characteristics of domestication syndromes that are evident in most plant and animal species. Indeed, Rokas (2009) speculated that a lack of relaxed selection acting on some domesticated microbes may reflect fundamental differences in population dynamics of microbes.

*Aspergillus oryzae* (Ahlb.) (*Ascomycota*, *Eurotiales*) is thought to have been domesticated thousands of years ago. The solid-state cultivation techniques used to culture this fungus date back 2,000–3,000 years ago (Machida et al. 2008) and the first commercial sales of Koji (rice fermented with *A. oryzae*) are evident in the 13–15^th^ century (Machida et al. 2008). Early descriptions of *A. oryzae* listed it as a variety of *Aspergillus flavus* Link (Wehmer 1901), but by 1921, it was recognized as a separate species (Thom and Church 1921). Molecular evidence strongly supports that *A. oryzae* was domesticated from *A. flavus*, a plant and animal pathogen notorious for its production of the potent carcinogen, aflatoxin. Gibbons et al. (2012) clarified that *A. flavus* isolates span a spectrum of relatedness to *A. oryzae* and suggested that domestication likely occurred from an atoxigenic lineage of *A. flavus*.

Despite this relationship, the taxonomic status of *A. oryzae* remains debated. Recent taxonomic treatments have alternatively regarded *A. oryzae* as a domesticated form of *A. flavus* retained as a separate name because of its industrial importance, while continuing debate surrounds its taxonomic status (Frisvad et al. 2019; Houbraken et al. 2020; Visagie et al. 2025). Recent phylogenomic analyses have further complicated this picture, suggesting admixture and multiple domestication-associated lineages within the broader *A. flavus*–oryzae complex, particularly involving closely related populations (Pfliegler et al. 2025). Throughout this study, we use *A. oryzae* as an operational designation for isolates belonging to historically recognized *A. oryzae* lineages identified in previous genomic studies (e.g., Watarai [2019]). These lineages include both industrial and closely related environmental isolates and provide a consistent framework for examining evolutionary patterns associated with domestication without making assumptions regarding the ultimate taxonomic boundaries of *A. oryzae*.

The population structure of *A. flavus* has also undergone substantial revision. Geiser et al. (2000) originally divided *A. flavus* into lineages IA, IB, and IC. A subsequent global multilocus analysis of 1,304 isolates further highlighted extensive diversity within the species complex and identified several major genetic clusters within this framework (Moore et al. 2017). Using whole-genome data, Drott et al. (2020) demonstrated that the original lineage assignments did not map cleanly onto genome-wide population structure and proposed a revised framework defining genetically isolated populations A, B, and C based on population structure, phylogenetic relationships, and allelic variation. Population A and B were widespread and largely sympatric, whereas population C was predominantly associated with northern latitudes and contained relatively few aflatoxin-producing isolates. Subsequent genomic and pangenomic studies generally recovered similar structure, although direct correspondence between newly identified groups and previous nomenclature has not always been established (Gangurde et al. 2024). More recently, analysis of Asian and European clinical isolates identified a fourth population, population D, that is significantly enriched for clinical strains (Hatmaker et al. 2025). Pangenomic analysis by Xie et al. (2026) further suggested substantial differentiation of gene content and putatively adaptive metabolic profiles within some populations, suggesting the resolution of subclades within population A. Recent phylogenomic analyses incorporating *A. oryzae* have further complicated this picture by identifying evidence of admixture between groups, particularly amongst *A. oryzae* and populations C and D, raising the possibility of multiple domestication events within the broader *A. flavus*–oryzae complex (Pfliegler et al. 2025).

Loss of aflatoxin production among isolates of *A. oryzae* has been extensively studied and has multiple etiologies (Tominaga et al. 2006; Kiyota et al. 2011). Balancing selection for aflatoxin production (Carbone et al. 2007; Drott et al. 2017, 2019b) is thought to maintain closely related aflatoxigenic and non-aflatoxigenic lineages. The fact that aflatoxigenic and non-aflatoxigenic isolates are often found at similar prevalences in the same geographic area (Drott et al. 2020) raises a question of how this organism could have been domesticated without many poisonings (although this human cost could have been lost to history). Intriguingly, the spore density of *A. flavus* decreases dramatically at higher latitudes (Drott et al. 2019a), and there is evidence that population C is largely northern (Xie et al. 2026), although sampling in the southern hemisphere is lacking. In contrast to other populations, population C is also disproportionately non-aflatoxigenic (Drott et al. 2020; Xie et al. 2026). Notably, Japan, where Koji was first produced (Machida et al. 2008) commercially, is predominantly northern. To clarify the impact of domestication on *A. oryzae*, there is a need to examine extant relatives that more closely reflect the lineage originally used for domestication.

Microbes, just like plants and animals, are domesticated through artificial selection of desired traits. In contrast to macroscopic organisms, selection on microbes is typically imposed for their chemical diversity. Gibbons et al. (2012) found selection in *A. oryzae* acting on flavor-related glutaminase genes and revealed an increased copy number of amylase genes. Critically, artificial selection would also strongly select against toxic metabolites. A hallmark of the domestication of *A. oryzae* has been a dramatic downregulation of several secondary metabolite (SM) gene clusters that often encode toxic compounds (including aflatoxin) (Gibbons et al. 2012). Transcriptomic rewiring is thought to be a major mechanism of phenotypic differentiation among even very closely related species, e.g. humans and other apes (Gould 1977; Johnson 2017). The expression of *Aspergillus* SMs is impacted by ecological factors that domestication would have shifted, including nutrient environment (Georgianna et al. 2010) and spore density (Affeldt et al. 2012). Density-dependent transcriptomic rewiring is known to suppress aflatoxin production at higher densities (Affeldt et al. 2012). Nearly 25% of the pan-secondary metabolome in *A. flavus* is differentiated between populations (Drott et al. 2021). However, almost all past transcriptomic work in *A. flavus* has focused on a single reference genome, leaving questions about the population-transcriptomic patterns that may clarify the domestication of *A. oryzae*.

To investigate the molecular basis of population-level variation, we conducted a comparative transcriptomic analysis of *A. flavus* and *A. oryzae*. Specifically, we sought to determine whether: (i) transcriptomic profiles are structured by population-specific differentiation; (ii) distinct populations exhibit divergent responses to density-dependent metabolic rewiring; (iii) forces beyond phylogenetic relatedness have shaped the evolution of transcriptomic architectures; and (iv) population-specific transcriptional programs can be attributed to a discrete set of regulatory factors. We also examine variation in pathogenic potential among isolates, including domesticated lineages. Together, these analyses suggest candidate mechanisms by which regulatory divergence contributes to metabolic diversification in *A. flavus*, providing new insights into the domestication trajectory of *A. oryzae*.

## Methods

### Population sampling and genome sequencing

We sampled five to seven isolates from the three *A. flavus* genetically isolated populations (A, B, and C) that were known at the inception of this project (Drott et al. 2020). A fourth genetically isolated population of small-sclerotia producing isolates (S-type) was also confirmed by Drott et al. (2020) but has been treated separately from large-sclerotia (L-type [populations A, B, and C]) here and elsewhere in the literature (Geiser et al. 2000; Drott et al. 2019a; Hatmaker et al. 2025) because of extreme phenotypic differentiation in both colony morphology and aflatoxin production (e.g., S-type produce almost no conidia and all isolates produce very high amounts of aflatoxin). From each population, a mixture of aflatoxigenic and non-aflatoxigenic isolates were selected as evenly as possible (Suppl. material 1: table S1) while sampling broadly from across each population’s clade (based on results from Drott et al. (2021)). An additional six *A. oryzae* isolates (Suppl. material 1: table S1) were collected from the public and the author’s culture collections (Drott collection currently housed at USDA-ARS Cereal Disease Laboratory). *A. oryzae* isolates were similarly chosen to be as genetically and geographically as diverse as possible, although little information was typically available for isolates and industrial strains are rarely shared. Of these, two (RIB40 and NRRL506) already had genome sequences available. We define *A. oryzae* based on affiliation with historically recognized *A. oryzae* lineages identified in previous genomic studies (e.g., Watarai et al. [2019]), including both industrial and closely related environmental isolates. Isolates were selected to sample broadly across the diversity represented within these lineages, providing a consistent framework for transcriptomic comparisons while remaining agnostic regarding the ultimate taxonomic status and evolutionary boundaries of *A. oryzae*. We sequenced the genomes of the remaining four isolates using methods similar to Drott et al. (2020) using the Illumina NovoSeq 6000 platform. Resulting sequence data was used to call SNPs using the GATK (Van der Auwera and O’Connor 2020) pipeline as defined by Drott et al. (2020). Resulting SNPs were integrated with data from Drott et al. (2020) and used to construct a neighbor-net network in SplitsTree v6.3.31 (Huson and Bryant 2006) to confirm that isolates used in the study were not clones (see methods below). While NRRL506 shared recent common ancestry with RIB40, it was retained given the distant isolation locations of the two isolates and questions about the importance of clonality in this domesticated lineage (Suppl. material 2: fig. S1). A final list of selected isolates is available in Suppl. material 1: table S1.

### Acquisition of public genomes, annotation, and reference selection

All genomes of *A. flavus* and *A. oryzae* that were available on NCBI were downloaded on 5/24/22 (a list of accessions used here are available in Suppl. material 1: table S2). Genomes were annotated with AUGUSTUS v3.3.2. with pre-built *A. oryzae* gene models (Stanke and Morgenstern 2005). A chromosome assembly of the *A. flavus* reference genome, NRRL3357 (Aspfl2_3), and the associated genome-annotation that was originally developed in Drott et al. (2020) was obtained from JGI and used as a reference genome.

### Pangenome construction

We organized predicted proteins from our total genomic dataset into orthogroups using OrthoFinder v2.5.5 (Emms and Kelly 2019). Functional annotations of proteins in orthogroups were identified from PFAM domain-associated GO terms using InterProScan v5.44-79 (Jones et al. 2014). Orthogroups found in all genomes were considered core. While this cutoff for ‘core’ sequences is more stringent than many pangenomic studies (often using 95%), this approach allows us to interpret expression patterns within the core genome without concern that they are influenced by even low-frequency presence-absence polymorphisms. Although we used pan-genomic inference to inform our transcriptomic analysis, more comprehensive analyses of the *A. flavus* pangenome have recently been conducted by Hatmaker et al. (2025) and Xie et al. (2026).

### Analysis of SM gene clusters

Biosynthetic gene clusters (BGCs) were identified from genome assemblies using antiSMASH v5.1.2 (Blin et al. 2019). While newer versions of antiSMASH have incorporated some new protocluster types, including the recently defined class of isocyanides (Nickles et al. 2023), these versions were released after our formal analysis had concluded. These new protocluster types are relatively rare in *A. flavus* (e.g., the newest version finds one isocyanide cluster in the NRRL3357 genome, although we note that Nickles et al. (2023) found two [https://isocyanides.fungi.wisc.edu/bgcs.html?accession=GCA_000006275.3&species=Aspergillus_flavus]). Furthermore, antiSMASH is prone to border-call errors whereby clusters are predicted to be too large, too small, or multiple clusters are called together. Even the newest versions of this algorithm still miss some known clusters (e.g., Kojic acid). In our approach, we manually curated borders of 15 characterized SMBGCs and the sequences of population-specific BGCs from Drott et al. (2021). We suggest that our approach is robust and while patterns we observe may extend to new clusters, the addition of a small number of protocluster classes is unlikely to change our overarching conclusions. The relationship of BGCs showing population-specific variations were identified and compared to Drott et al. (2021) using BLASTp v2.12.0+ from the BLAST+ suite (Camacho et al. 2009). We dereplicated BGCs by creating networks with Biosynthetic Gene Similarity Clustering and Prospecting Engine (BiG-SCAPE) (Navarro-Muñoz et al. 2020), which were visualized with Cytoscape v3.7.1 (Su et al. 2014). Those BGCs that did not map to the reference genome or to other BGCs defined in Drott et al. (2021) are called “combos”. A table of clusters mapped to the reference genome is available in Suppl. material 1: table S3.

### Phylogenetic tree reconstruction

The original sampling of isolates was based on population structuring inferred by Drott et al. (2020). To confirm these relationships in the presence of public data, we reconstructed phylogenetic trees from assembled genome sequences. Benchmarking single copy ortholog (BUSCO) sequences corresponding to the fungal database v10 were obtained using BUSCO (Simão et al. 2015). Maximum likelihood phylogenies of BUSCO sequences were constructed by aligning sequences with MAFFT (Katoh et al. 2002), trimming with trimAL (Capella-Gutiérrez et al. 2009), and using the test parameter across a partition model in IQ-TREE2 (Nguyen et al. 2015) with pre-filtering similar to previous work (Steenwyk et al. 2019) as is detailed in the Suppl. material 2: supplemental methods. Phylogenetic trees were also constructed for each of the genes in the EurotialesBUSCO dataset and used to construct a quartet-based tree in ASTRAL (Zhang et al. 2018). We also constructed phylogenies from SNP data using IQ-TREE2 with methods identical to those used previously (Drott et al. 2021). Additionally, SNP data from Hatmaker et al. (2025) was integrated with data from this study using IQ-TREE2, as detailed in the Suppl. material 2: supplemental methods. Resulting trees were visualized using ggtree (Yu et al. 2017) and iTOL v6 (Letunic and Bork 2024), respectively.

To clarify the relationship between *A. flavus* and *A. oryzae* considering recent inferences suggesting that these taxa may not be each other’s closest relatives (Kjærbølling et al. 2020), we downloaded all *Aspergillus* reference genomes available from NCBI on February 8, 2026 (n = 174). Phylogenetic analyses were constructed using protein sequences of 3,450 conserved eurotiomycete BUSCO genes present in single copy in at least 95% of genomes (inferred in genome mode). Gene alignments and trimming were performed as described in the Suppl. material 2: supplemental methods for our other phylogenetic analyses, and trees were inferred using IQ-TREE2.

To further evaluate species identities reported on NCBI, we additionally analyzed genomes assigned to *Aspergillus minisclerotigenes* Vaamonde, Frisvad & Samson, *Aspergillus aflatoxiformans* Frisvad, Ezekiel, Samson & Houbraken, and *Aspergillus austwickii* Frisvad, Ezekiel, Samson & Houbraken, which are listed in Frisvad et al. (2019) as among the closest relatives of *A. flavus* and *A. oryzae*. To integrate these data, we used *A. oryzae* sequences for ITS (EF661560), BenA (EF661483), CaM (EF661506), and RPB2 (EF661438) as BLAST queries to extract corresponding sequences from both NCBI reference genomes and the isolates included in this study. Resulting sequences were aligned, trimmed, and phylogenies were inferred using an edge-linked partition model in IQ-TREE2, following the same procedures described in the Suppl. material 2: supplemental methods for our other phylogenetic analyses.

### Clone correction

To collapse genetically identical individuals, we performed clone correction similar to DeGenring et al. (2025). Briefly, the ‘mldist’ data matrix output by IQ-TREE2 from the BUSCO maximum likelihood tree was reformatted into a list of pairwise comparisons between isolates using a custom script that uses the R package *reshape2* v1.4.4 (Wickham 2007). The resulting pairwise distances were visualized as a histogram. We assumed that the peak and tail at/near zero genetic distance represents true clonal relationships, and a distance cutoff of 5.50 × 10^–6^ was selected to capture this near-zero distribution (Suppl. material 2: fig. S2). When isolates shared a pairwise distance less than our cutoff, they were collapsed into a single multi-locus genotype (MLG, Suppl. material 1: table S2, Suppl. material 2: fig. S2). When clone-corrected data were used, the genome with the highest BUSCO completeness metric was selected to represent an MLG. This approach is flexible across datasets, allowing for data-driven and transparent clone correction of diverse datasets.

### Experimental design and population transcriptomic sequencing

To dissect the transcriptomic differentiation of *A. flavus* and *A. oryzae* populations, we chose an experimental design that favored biological replication within a population instead of technical reps, using five to seven isolates from each population as biological replicates within a population (Suppl. material 1: table S1).

Fungal spores were harvested from colonies growing on PDA using 0.05% tween 20 water. Resulting spore suspensions were diluted to either 10^2^ or 10^6^ spores/ml and a 200 ul suspension was then spread evenly across a piece of sterile cellophane resting on a PDA. Spore concentrations were selected in line with previous reports of density-dependent shifts in colony morphology and aflatoxin production (Brown et al. 2009a; Affeldt et al. 2012). Concentrations were adjusted slightly lower, from 10^3^ and 10^7^ used in previous work, to better reflect spore densities found in agricultural soils( ~10–2,500 CFU/gram found by Drott et al. [2019a]), and optimal densities for industrial koji production (5 x 10^5^ according to Kusumoto et al. [2021]). We speculate that micro-scale spore concentrations may well fully cover this range and emphasize that on a macroscale, spore densities vary across latitudes (Drott et al. 2019a) with some genetic populations changing in prevalence across this gradient (e.g., population C is more common at higher latitudes Drott et al. [2020] and Xie et al. [2026]). After 100 h, mycelia were collected, frozen in liquid nitrogen, and lyophilized for 24 h. Two plates for each condition were harvested, combined, and extracted using trizol (Ambion, USA), according to the manufacturers protocol. The purity and integrity of RNA were tested via gel electrophoresis, nanodrop, and in the Agilent 2100 bioanalyzer. The concentration of RNA samples were quantified precisely by Qubit 2.0. Libraries were prepared using the poly-A mRNA enrichment protocol and paired-end sequencing in the NovaSeq 6000 platform.

### Identification of differentially expressed genes

The transcriptomic read count of each gene was quantified with kallisto ver. 0.44.0 (Bray et al. 2016) using the NRRL3357 (Aspfl2_3) reference genome. Because the primary objective of this study was to investigate population-level differences in transcriptomic architecture, we chose to focus on the portion of the genome that could be consistently compared across groups (core genome). However, this approach may underestimate the contribution of accessory genes by representing only those accessory genes present in the reference genome. While this design was chosen to facilitate interpretation, we note that it ignores population-specific differences among accessory genes that are not present in the reference. A Wald test from the R package DESeq2 (Love et al. 2014) was used to identify the differentially expressed genes between spore density for each population.

### Co-expression network

Weighted Gene Co-expression Network Analyses (WGCNA) were used to identify co-expressing gene modules at the population level. First, the read counts were normalized with Trimmed Mean of the M-values (TMM) and Fragments Per Kilobase per Million mapped fragments (FPKM) using the R package *edgeR* (Robinson et al. 2010), with special attention paid to the default parameter that weights gene-entry order. Next, a weighted gene co-expression network was constructed using the R package *WGCNA* with the log values of TMM-FPKM (Langfelder and Horvath 2008). The dendrogram cut height to merge modules was set to 0.25 and the soft threshold was set to 12. To understand the putative functions of these gene modules, the R package topGO was used to identify the gene ontology (GO) terms enriched in each module (Alexa and Rahnenfuhrer 2010).

### Phylogenetic analysis of transcriptomics

To test if genes were differentially expressed in different populations, we used three strategies: (1) PERMANOVA, (2) phylogenetic ANOVA and (3) two-way ANOVA. The function *adonis2* from the R package *vegan* (Dixon 2003) was used to perform a PERMANOVA to test if genome-wide gene expression level (log-TMM-FPKM) is dependent on population, spore density, toxicity, or any interacting terms. The function *aov.phylo* from the R package *geiger* (Pennell et al. 2014) was used to perform a phylogenetic ANOVA focusing on a single spore density at a time to separate the effects of the independent variables between the two treatments. Two-way ANOVAs were done with base R using population, spore density, and their interacting term as independent variables and gene expression level as the dependent variable. The p-values from ANOVA were further adjusted again using False Discovery Rate (FDR) for the number of genes tested. The analyses were also done on gene expression modules (normalized expression value) and the backbone genes of secondary metabolism clusters (log-TMM-FPKM; mean of log-TMM-FPKM if multiple backbone genes were identified), but the toxicity term and any associated interaction terms were not included in the PERMANOVA.

### Gene Regulatory Network (GRN) inference and analyses

Normalized read counts from the co-expression network analysis were used as inputs to SC-ION v3.2 (https://github.com/nmclark2/SCION) (Clark et al. 2021), which employs a modified version of the tree-based regression method GENIE3 (Huynh-Thu et al. 2010). Networks were inferred separately with a random seed of “11111” to allow for replication, using only differentially expressed genes from each of the condition, population, and condition*population contrasts (adj. *p* < 0.05) based on a two-way ANOVA analysis (Suppl. material 1: table S6). Transcription factors annotated in MycoCosm (https://mycocosm.jgi.doe.gov/Aspfl2_3/Aspfl2_3.home.html) were assigned as potential regulators, and all differentially expressed genes were assigned as potential targets. The resulting GRNs were trimmed to remove edge weights < 0.65. This cutoff corresponded to the best supported 0.28%, 1.31%, and 16.04% of edges in the population, spore density, and population * density contrast networks, respectively. GRNs were analyzed using the *igraph* and *influential* R packages (Csardi and Nepusz 2006), and node centrality was calculated using the Integrated Value of Influence (IVI) metric (Salavaty et al. 2020). Networks were visualized in Cytoscape version 3.10.2 (Shannon et al. 2003).

### Zebrafish husbandry and maintenance

Wild-type AB Zebrafish (*Danio rerio* [Hamilton, 1822]), the common wild-type obtained from ZIRC (https://zebrafish.org/home/guide.php), lines were maintained under standard conditions as described previously (Knox et al. 2014). For experiments, embryos were collected and maintained at 28.5 °C in E3 water.

### Fungal inoculum preparation and microinjections

Fungal strains were grown from glycerol stocks on Glucose Minimal Media (GMM), and spores were harvested and prepared as previously described (Schoen et al. 2021, 2023). The conidial concentration was adjusted to 1.5 x 10^8^ conidia/mL and then mixed 2:1 with 1% phenol red before injection. Larvae were anesthetized at 2 days post-fertilization and injected with 3 nL of conidial suspension into the hindbrain ventricle. After injections, larvae were rinsed three times with E3 and transferred to individual wells in 96-well plates. Survival was monitored for 7 days. After injections, eight larvae per condition were transferred to individual microcentrifuge tubes containing 90 μL 1X PBS with 500 μg/mL kanamycin and 500 μg/mL gentamycin. Larvae were homogenized for 15 seconds in a mini bead beater, plated on GMM, and incubated for 2 days at 37 °C. After incubation, colony-forming units (CFUs) were counted and averaged for each condition. The pairwise log-rank test comparisons in larval survival were calculated using the R packages survival v3.8-3 (Therneau 2015) and survminer v0.5.0 (Kassambara et al. 2016), using the pairwise_survdiff() command, with Benjamini-Hochberg adjustment for false-discovery rate correction.

## Results

### *A. oryzae* shares recent common ancestry with *A. flavus* population C and overlaps with a clinically enriched lineage

Phylogenetic analysis of publicly available genomes from NCBI indicated that the reference assemblies labeled as *A. austwickii* (GCA_037043805.1) and *A. aflatoxiformans* (GCA_037043995.1) are separated from *A. flavus* by branch lengths comparable to those observed between *A. flavus* and *A. oryzae* (Suppl. material 2: fig. S3). However, when evaluated in the context of the taxonomic framework proposed by Frisvad et al. (2019), these genomes cluster within the broader *A. flavus* clade rather than clustering with their respective species (Suppl. material 2: fig. S4). This pattern is consistent with prior reports of taxonomic ambiguity and potential misidentification within section *Flavi* (Steenwyk et al. 2024) and beyond (Houbraken et al. 2021). Across all analyses conducted here, we find no evidence supporting the presence of a closer relative to *A. flavus* than *A. oryzae* within currently available genomic data.

To clarify the domestication origins of *A. oryzae*, we reconstructed phylogenetic relationships from genome-wide SNP datasets including all 793,820 biallelic SNPs, 629,271 SNPs with no missing data, 430,178 intergenic SNPs, and a thinned (to 1 kb distance) subset of 20,067 intergenic SNPs (Suppl. material 2: fig. S5). We also constructed a neighbor-net network to better infer potential recombination as reticulation (Suppl. material 2: fig. S1). All phylogenetic methods consistently offered strong support that *A. oryzae* shares the most recent common ancestry with population C of *A. flavus*, a largely northern and non-aflatoxigenic lineage. Branch lengths in these analyses are consistent with our treatment of *A. oryzae* as analogous to an *A. flavus* population. Trees built using BUSCO sequences to enable the incorporation of public gene presence/absence data (discussed below in this section) were sometimes unstable and/or gave different topologies (Suppl. material 2: figs S6, S7). Because BUSCO loci are highly conserved, SNP-based datasets provided greater resolution for intraspecific relationships and were therefore used to infer population structure. Overall, these results support a most recent common ancestry between *A. oryzae* and population C of *A. flavus*.

**Figure 1.**
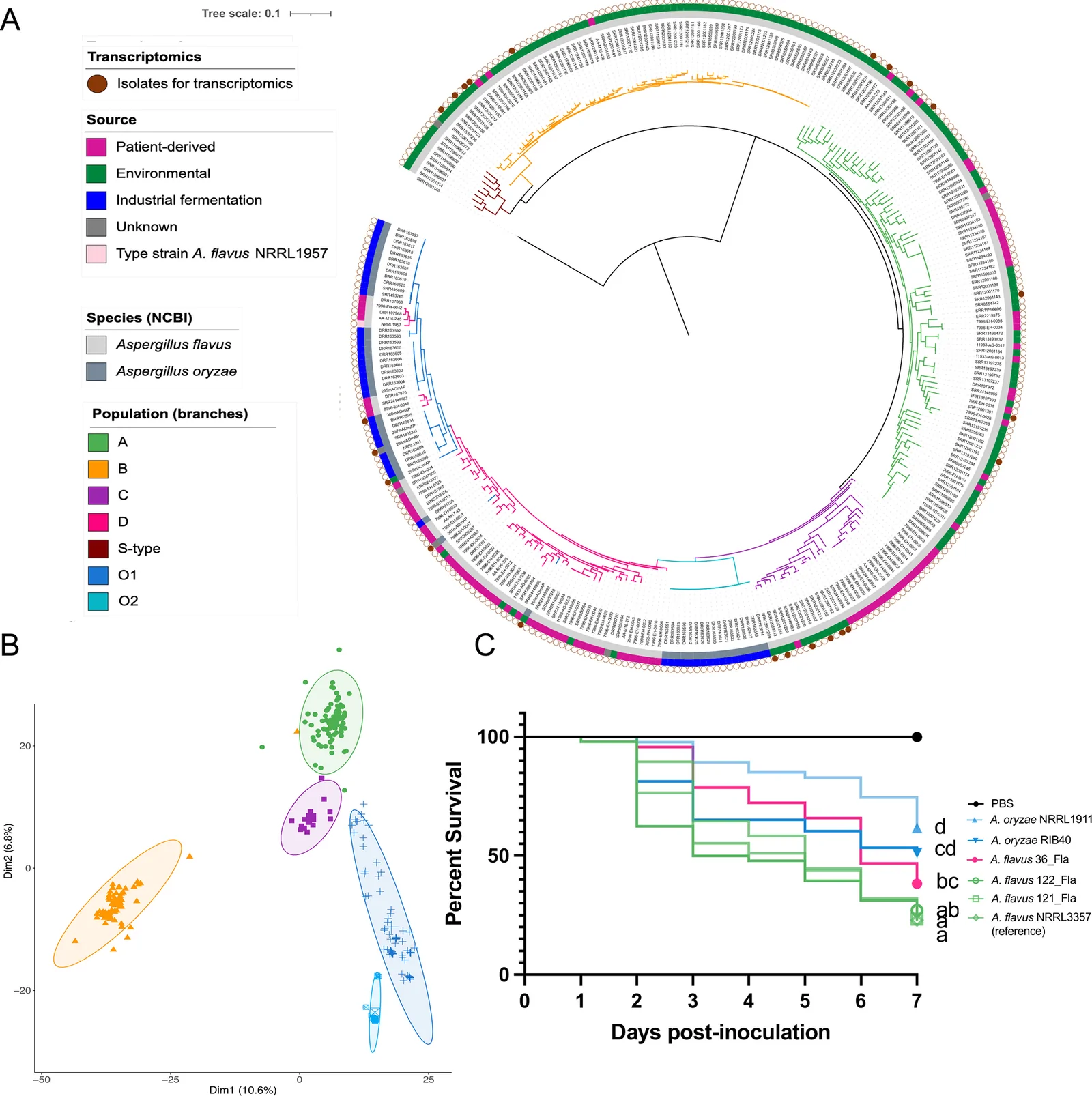
*Aspergillus oryzae* shares recent common ancestry with *Aspergillus flavus* population D and population C. **A** A maximum likelihood phylogeny constructed using genome-wide SNPs including data from Hatmaker et al. (Hatmaker et al. 2025) is rooted at *Aspergillus minisclerotigenes*. Bootstrap supports for this tree are depicted in fig. S8. **B** principal component analysis of *A. flavus* isolates across orthogroups showing the relationship in isolates and populations based on gene-space. Colors correspond to populations defined in A. **C** Kaplan-Meier survival curve of Zebrafish assay demonstrating that *A. flavus* isolates from population A and D, and *A. oryzae* all show virulence in an animal model compared to the PBS control. Lines are colored in shades that correspond to populations defined in panel A. Letters indicate statistical significance groups (log-rank test, *p* < 0.05, Benjamini-Hochberg adjusted p-values).

To place our results in the context of recently recognized diversity within *A. flavus*, we incorporated population D, a lineage enriched for clinical isolates identified by Hatmaker et al. (2025), into our phylogenetic analyses. We integrated SNP data from our *A. oryzae* isolates into the Hatmaker dataset and found that *A. oryzae* isolates were interspersed within population D (Fig. 1A). The inclusion of population D in the phylogeny subdivides the *A. oryzae* clade from a monophyletic to a polyphyletic structure, suggesting multiple domestication events and/or recombination. This reanalysis also found that population D and *A. oryzae* were strongly supported as most closely related to population C, the same topology as we identified above (Suppl. material 2: fig. S8). The genomes of population D isolates are significantly larger than those in all other populations, perhaps reflecting a recent expansion (Suppl. material 2: fig. S9).

Principal component analysis of gene presence/absence data supported the phylogenetic results, with *A. oryzae* grouping most closely with population C (Fig. 1B); PC1 explained 10.6% of the total variance and PC2 explained 6.8%. Subsequent components each explained progressively smaller proportions of variance (PC3: 5.0%; PC4: 3.1%; PC5: 2.9%). In total, 17 principal components were required to explain 50% of the variation. Functional enrichment among differentially present genes has recently been addressed by Hatmaker et al. (2025); here we find enrichments include categories related to reproduction, viruses, and transposable elements (Suppl. material 2: supplemental results).

Clonality varied widely across populations: A (27.5%), B (43.7%), and C (26.7%). In contrast, *A. oryzae* showed a higher overall clonal fraction (75.2%) and split into two major clades—designated O1 and O2 — with clonal fractions of 66.7% and 97%, respectively (Suppl. material 1: table S2, Suppl. material 2: fig. S2). Both clades contained industrial strains, but only O1 included environmental isolates. The O2 clade was composed of just two genetically distinct multi-locus genotypes (Suppl. material 2: figs S2, S4).

### *A. oryzae* retains pathogenic potential in a Zebrafish model

Given the close relationship between clinically enriched population D *A. flavus* isolates and *A. oryzae*, we assessed pathogenic potential using a Zebrafish model of aspergillosis (Fig. 1C) to evaluate whether domesticated lineages or closely related clinical populations retained virulence. We examined two *A. oryzae* reference isolates (RIB40 and NRRL1911), the *A. flavus* reference strain NRRL3357 (population A), and three *A. flavus* clinical isolates (121_Fla and 122_Fla [population A] and 36_Fla [population D]). All isolates caused host mortality under the conditions tested.

While our sampling is insufficient for population-level conclusions, survival analyses revealed significant isolate-level variability. Both population A clinical isolates exhibited the highest mortality rates, whereas the population D isolate and both *A. oryzae* isolates showed comparatively lower mortality patterns. Specifically, population A isolates differed significantly from NRRL1911 (*p* < 0.0001) and RIB40 (*p* = 0.028), and from the population D isolate (*p* = 0.047). The population D isolate differed significantly from NRRL1911 (*p* = 0.028) but not from RIB40. The environmental population A isolate, NRRL3357, did not display a significant difference in virulence from clinical isolates of the same population (Fig. 1C). While variation in microinjection inoculum load is unavoidable and could influence survival outcomes, we found no correlation between inoculum load and virulence across strains (*p* = 0.939, Suppl. material 2: fig. S10). This suggests that the observed differences in mortality likely reflect genotype-specific differences and cannot be explained solely by variation in inoculum load.

Although our sampling does not permit definitive species-level conclusions, these results suggest a population-level trend in which isolates within or closely associated with the population D clade, including *A. oryzae*, exhibit lower pathogenic potential relative to certain population A clinical isolates. We interpret these findings as contextual evidence inconsistent with the hypothesis that clinical enrichment within the population D clade universally corresponds to elevated virulence in this or closely related domesticated lineages. While our results provide a preliminary risk assessment, broader strain sampling will be required to formally define population- and species-level virulence differences.

### Population transcriptomics reveal population-specific expression profiles

**Figure 2.**
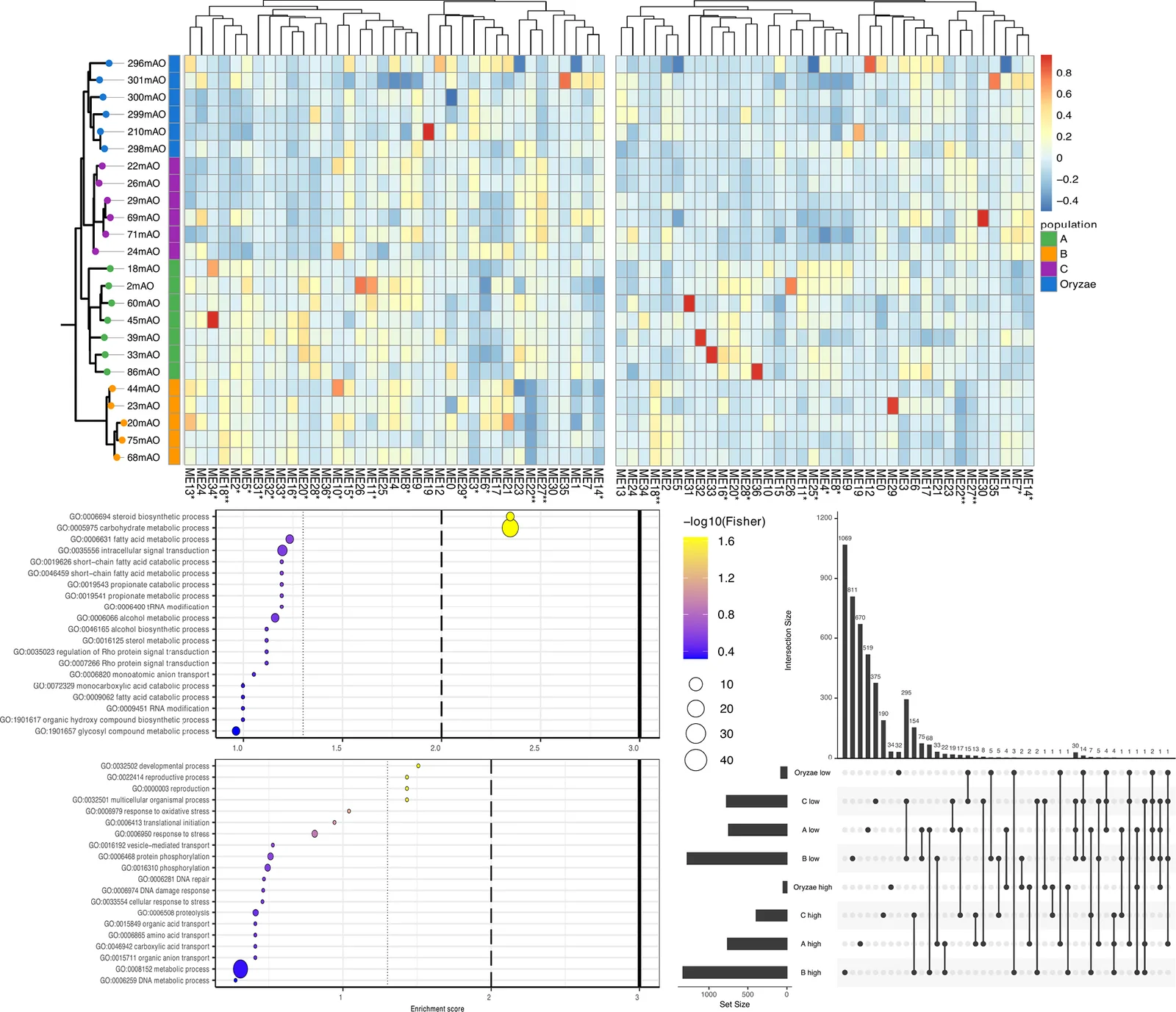
*Aspergillus flavus* and *Aspergillus oryzae* populations differentially express genes and modules as a function of spore density. **A** Heatmaps depicting the expression of module eigenvalues obtained from Weighted Gene Co-expression Network Analyses across isolates grown at low (left) or high (right) density. One star on the module labels represents significant differentiation between populations (ANOVA) while two stars indicate significance when accounting for phylogenetic distances (phylogenetic ANOVA). Isolates of *A. flavus* and *A. oryzae* populations are organized according to a maximum-likelihood phylogeny constructed from the genetic relationships of 173 high-quality BUSCO genes. Hierarchical clustering based on combined density data is presented above both heatmaps. **B** Two examples of the GO enrichment biological process terms present in significantly differentially expressed modules are given for module ME15 (top) and ME22 (bottom). The three increasingly solid dotted lines on enrichment plots indicate significance at the 0.05, 0.01, and 0.001 levels, respectively. **C** The differential expression of individual genes is represented in an upset plot that depicts which differentially expressed genes (DEGs) are more highly expressed in a given spore density (e.g., there are 295 DEGs shared between pops B and C that are expressed more in the low spore density and 154 genes that are shared between these pops at the high spore density, for a total of 449 density-dependent DEGs shared between these populations).

We examined whether gene expression varied significantly between populations and how this variation was influenced by an ecologically relevant factor: spore density, which influences SM production and fungal development (Brown et al. 2008), and varies across geographic regions and industrial and agricultural environments (Drott et al. 2019a; Kusumoto et al. 2021; Xie et al. 2026). Gene expression and gene module expression were both significantly differentiated among populations (PERMANOVA, *p* < 0.001, Suppl. material 1: table S4 and S5 respectively), spore density (*p* = 0.005) and their interaction was significant across genes (*p* = 0.022) and modules (*p* = 0.029). Aflatoxin producing ability did not have a significant impact on expression profiles. A total of 4,568 genes were differentially expressed among populations (two-way ANOVA, adjusted *p* < 0.05, Suppl. material 1: table S6). As expected, spore density also influenced transcriptomic profiles, but its effect was modest, with only 229 genes showing differential expression. Notably, the transcriptomic response to spore density of 73 genes varied significantly across populations (*p* < 0.05), suggesting population-specific plasticity. At low density, 22 modules were differentially expressed between populations (Suppl. material 1: table S7); 12 modules were differentially expressed at high density (ANOVA, unadjusted *p* < 0.05, Fig. 2, Suppl. material 1: table S8). Hierarchical clustering independent of population designations revealed that transcriptomic differentiation is not uniformly distributed across genetic clusters, but instead reflects modular, population-specific patterns (Suppl. material 2: fig. S11). While population-specific gene content may contribute to these findings, more than 70% of differentially expressed genes (DEGs) identified in our alignment to the reference — across population, condition, and their interaction — resided in the core genome offering robust validation of this patterning as similar studies have not performed tandem pangenomics and transcriptomics. However, we note that this analysis ignores accessory genes that are not present in the NRRL3357 reference genome; while the relatively large core genome used in this analysis offers simpler interpretation of overarching trends, future efforts using a pangenome-graph alignment approach could further clarify population-specific differentiation of the accessory genome.

Three co-expression modules (18, 22, and 27) showed significantly greater expression divergence than expected from phylogeny alone (phylogenetic ANOVA; adjusted *p* < 0.05, Suppl. material 1: table S8). Module 22, which had notably low expression in population B, was enriched for development and reproduction genes (Fisher’s exact test, unadjusted *p* = 0.031 and 0.037). Module 15 was more highly expressed across all groups compared to population A at low density (ANOVA, *p* = 0.040; phylogenetic ANOVA, *p* = 0.782). At high density, expression was highest in three *A. oryzae* isolates, though population-level differences were nonsignificant (ANOVA, *p* = 0.111). The latter module was enriched for carbohydrate metabolism genes (Fisher’s exact test, unadjusted *p* = 0.005; Fig. 2B).

Module 27 was marked by lower expression in *A. oryzae* and population B relative to all other populations (p < 0.0001). This module was enriched for nucleoside and glycosyl metabolism (p < 0.05) and amino acid transport (p < 0.01). Notably, industrial and environmental O1 isolates exhibited similar expression patterns within this module, differentiating them together from the major *A. flavus* populations. This result suggests that the historically recognized O1 lineage can behave as a biologically coherent transcriptomic unit despite broad geographic and ecological sampling. However, our transcriptomic sampling did not include clinical isolates from population D or industrial isolates from the O2 lineage. Future work incorporating these groups will be important for determining whether this transcriptomic coherence extends across the broader domestication-associated diversity now recognized within the *A. flavus*–oryzae complex and if any isolation-source specific patterns exist.

### Population and phylogenetic patterns of SM expression

**Figure 3.**
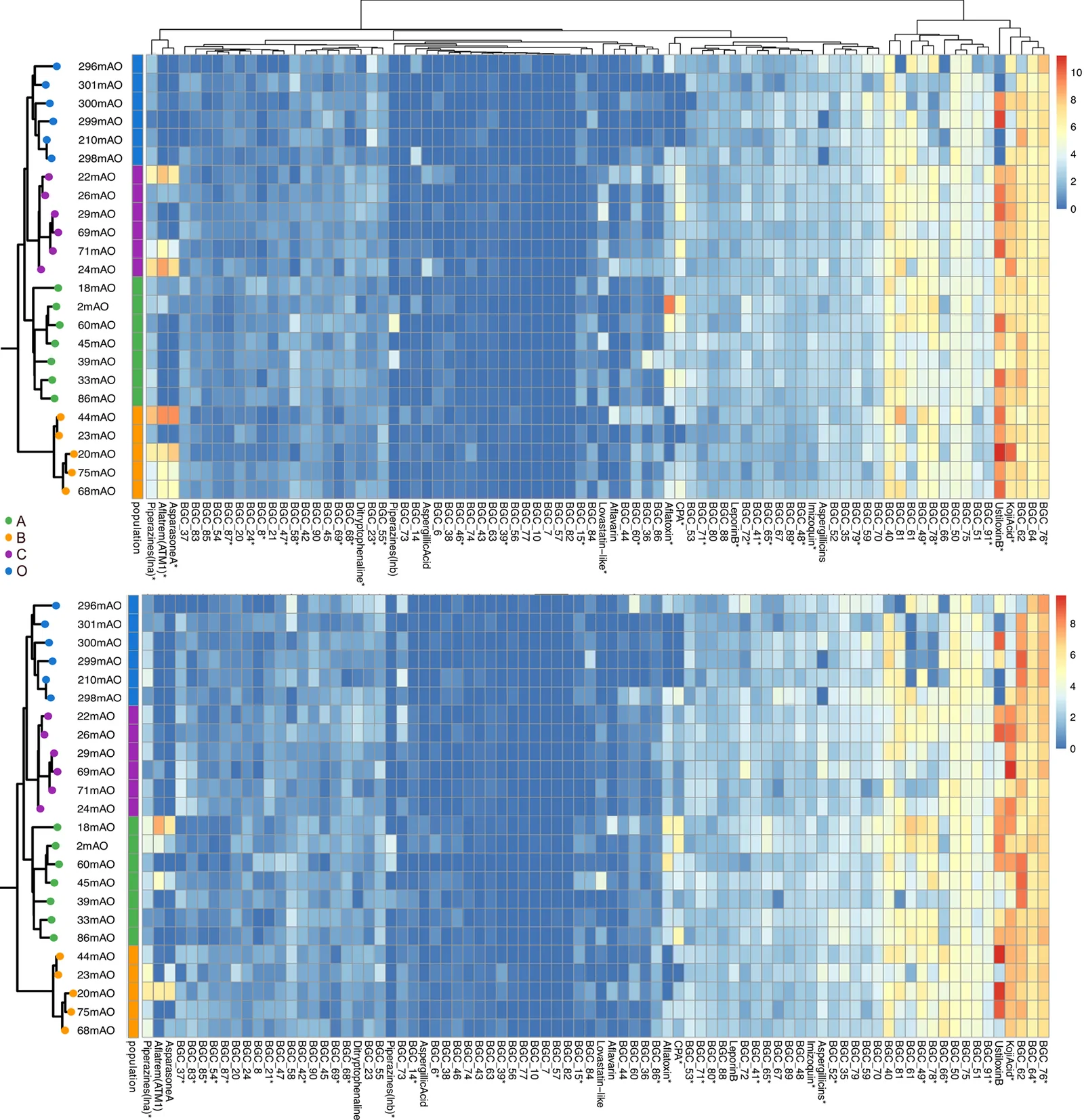
Heatmap depicting the normalized expression counts of secondary metabolite backbone genes that are present in the NRRL_3357 *Aspergillus flavus* reference genome as expressed at low (top) and high (bottom) spore density. Isolates of *A. flavus* and *Aspergillus oryzae* populations are organized according to a maximum-likelihood phylogeny constructed from the genetic relationships of 173 BUSCO genes. One star on the module labels represents significant differentiation between populations.

Previous work suggests that *A. oryzae* does not express certain highly toxic SMBGCs, including aflatoxin. To investigate the evolution of this phenomenon, we examined expression patterns of SM backbone genes across *A. oryzae* and three *A. flavus* populations. Overall, SM BGC backbone expression differed significantly between populations, densities, and weakly as an interaction between the two (PERMANOVA, *p* < 0.001, *p* = 0.005, and *p* = 0.059 respectively, Suppl. material 1: table S9). While differences in transcriptomic space across the entire genome were poorly defined in a principal component analysis of all genes, analysis of SM backbone genes identified a shift in the transcriptomic space of *A. oryzae* (Suppl. material 2: fig. S12). Hierarchical clustering revealed distinct expression clades: one clade (~15 backbones, rightmost in Fig. 3) was highly expressed across isolates and conditions; another, comprising three backbones — including asparasone A, aflatrem (ATM1), and a piperazine backbone (leftmost clade) — showed generally low expression, except in a small subset of isolates from populations B and C. The central clade had generally low expression with three subclades (from left to right): the first subclade showed low expression, the second subclade universally showed close to no expression and the final subclade showed intermediate expression, with aflatoxin and CPA forming a higher-expression group at the edge of this subclade (Fig. 3). Hierarchical clustering independent of population designations revealed that transcriptomic differentiation is not uniformly distributed across SM backbones but instead reflects modular patterns that are sometimes population-specific (Suppl. material 2: fig. S13). Given the common assumption that many BGCs are silent under laboratory conditions, it is notable that numerous backbone genes showed detectable levels of expression. However, transcript abundance does not necessarily imply active metabolite production. While our analyses demonstrate that expression patterns of certain BGCs differentiate populations, further work will be required to determine whether these pathways produce secondary metabolites, have been exapted for alternative functions, represent vestigial remnants of previously active biosynthetic programs, or reflect incomplete silencing. Importantly, even modest expression differences may have functional or fitness consequences — whether adaptive, neutral, or maladaptive — and distinguishing among these possibilities will require targeted genetic and chemical characterization.

Differences in expression between densities were evident for some BGCs. The aflatoxin backbone gene was most highly expressed in population A, particularly by NRRL3357 (isolate designation 2mAO — see Suppl. material 1: table S1) in low spore density, although there was variation within the population. The expression of ATM1, Asparasone A (both are core genes, Suppl. material 2: fig. S14) and Piperazines (lna) was high in populations B and C at low density, but not at higher density. Only isolates from population A showed medium–high expression of these genes at high density. The lovastatin-like gene cluster was significantly differentiated between populations at low density, but not at high density. Under both conditions, expression of this backbone was lower in *A. oryzae* and higher in population A.

### Population-specific SM BGCs

Because domesticated organisms often retain ancestral gene content, we examined the SMBGCs of *A. oryzae* in the context of broader *A. flavus* population diversity. We focused on differences between *A. oryzae* and *A. flavus* populations as comparisons within *A. flavus* populations were previously characterized by Drott et al. (2021).

*A. oryzae* retains nearly all BGCs found across *A. flavus* populations, reflecting its origin from within a large, shared core pangenome. Consistent with previous work, we do not find pervasive large-scale deletions of the aflatoxin gene cluster in *A. oryzae* (although some are evident), suggesting that inability to produce aflatoxin is often the result of SNPs, smaller indels, or regulatory changes. A subset of BGCs is found in low frequency or absent in *A. oryzae*, but these clusters are generally not fixed in the broader *A. flavus* population (e.g., BGC_5, Suppl. material 2: fig. S14).

Some population-specific BGCs appear in *A. oryzae* even when absent from other lineages. For example, the astellolide cluster, also found in population C, is present in some *A. oryzae* isolates. In other cases, population-specific BGCs occur at higher frequency in *A. oryzae* than in the population from which they were first observed (e.g., BGC_8, BGC_4, Suppl. material 2: fig. S14).

Several novel BGCs appear to be unique to *A. oryzae* but all of these occur at very low frequencies. The cluster dubbed ‘combo99’, for example, which shares 62% identity with auerobasidin A1 backbone across 73% of the protein from *Aureobasidium pullulans* (de Bary) G. Arnaud, is found in only a few *A. oryzae* genomes. Additionally, rare clusters that were previously excluded from population-level analysis of Drott et al. (2021) as singletons — such as combo100 and combo101 — are now included due to their presence in multiple *A. oryzae isolates*.

### Gene Regulatory Networks reveal differentiated transcriptomic architectures

To understand regulatory influences on transcriptional differences across conditions and populations, we constructed gene regulatory networks using significantly differentially expressed genes. Networks constructed with DEGs from condition and condition * population contrasts were small (Suppl. material 2: fig. S16). In the density network, only one regulator, 5739 (AFLA_044120), showed high centrality, targeting genes from BGC_90 and BGC_51 (Suppl. material 2: fig. S16B). In the condition * population contrast, 2210367 regulated 2207763 (BGC_47) and transcription factor 2283259 (BGC_52).

**Figure 4.**
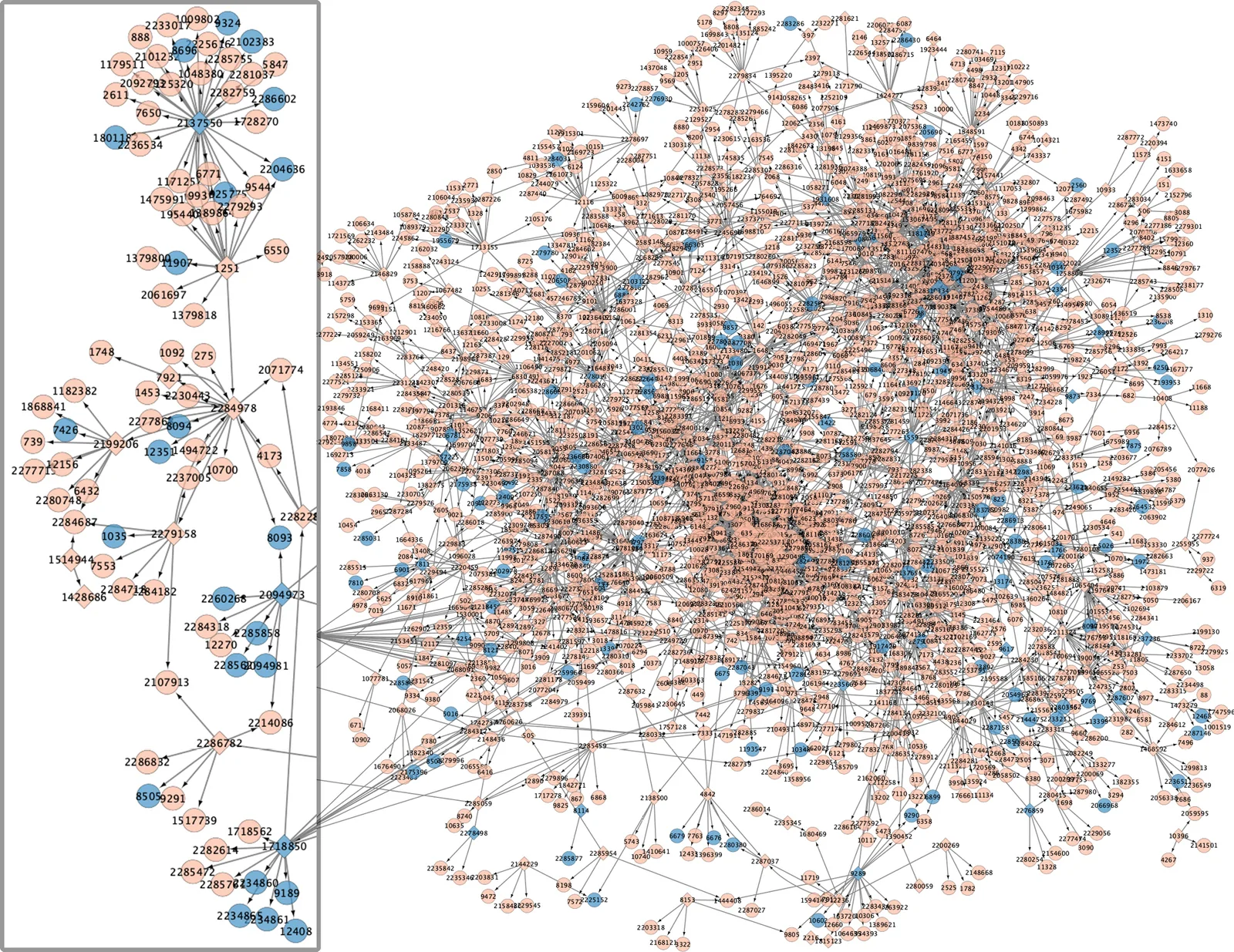
Gene regulatory network of genes that are significantly differentially expressed between *Aspergillus flavus* populations (**A–C** and *Aspergillus oryzae*). Nodes represent genes and are labeled with protein IDs from the NRRL3357 JGI Aspfl2_3 genome. Nodes corresponding to transcription factors are presented as diamonds while circles represent all other genes. Genes located in a secondary metabolism gene cluster predicted in the reference genome are colored blue. The size of nodes indicates the IVI centrality index, with larger nodes having higher centrality scores. Arrows indicate inferred regulatory direction. A portion of the network that shows regulatory interactions between BGCs is enlarged for clarity and is discussed in the text. A small number of genes from this network that shared no linkage with the main network displayed here were omitted for visual clarity but can be found in Suppl. material 2: fig. S15.

The population-based network included 2,177 nodes. The highest-centrality regulators were not strongly associated with BGCs (see lower-central-left mass in Fig. 4). For example, 2225371, the top-ranked transcription factor, regulated 160 genes, but only two (from BGC_67 and BGC_89) were BGC-associated. Regulators 635, 8311, and 2069970 were among the most central, sharing substantial target overlap, together forming 409 regulatory connections to 220 unique nodes, only three of which were in BGCs. This suggests large-scale population transcriptomic shifts are driven by regulators of primary rather than secondary metabolism. Supporting this, the global secondary metabolism regulator LaeA (1256857) was not differentially expressed.

In contrast, several lower-centrality transcription factors showed targeted regulation of secondary metabolism. Notably, 2137550 (AFL2G_00934.2, from the asparasone A cluster) regulated two aflatrem cluster genes — including the backbone (2204636) — in trans. It also targets genes from BGCs 35, 44, 57, and 85 (non-backbone; Fig. 4). The aflatrem backbone is also co-regulated by 1251 (AFLA_084720), and both 2137550 and 1251 coregulate a hypothetical gene in BGC85, with the former also regulating 1251, suggesting potential regulatory feedback. Interestingly, these interactions may help explain the co-expression pattern linking the asparasone A, aflatrem (ATM1), and piperazine (lna) backbones in Fig. 3, as 1251 also regulates the lna backbone gene (11907). Although the inferred interaction between 2137550 and the asparasone A backbone (2279316) fell just below our edge cutoff of 0.65 (0.644), its proximity to the threshold and the coordinated expression of these pathways are consistent with previous inferences of cis-regulation within the asparasone A cluster (Cary et al. 2014), in addition to the trans-regulatory relationships observed here.

Additional cis- and trans-BGC regulation included transcription factor 2094973 from the lovastatin-like cluster, which regulated three cluster genes (a P450, MFS transporter, and FAD monooxygenase; see callout in Fig. 4). This TF also putatively co-regulates a reductase and a beta-galactosidase in BGC24 (2285858 and 8093 respectively) with transcription factor 2284978 (AFLA_054800), which is itself downstream of 1251 and 2282289. Interestingly, 2282289 may also regulate the TF in the ustiloxin B cluster, 1718850, which controls six of the cluster’s genes (Fig. 4) in a population-specific way where *A. oryzae* is marked by lower expression. These results suggest that cluster-specific transcription factors can mediate trans-BGC expression, as has been found in several fungal species (Bergmann et al. 2010; Shostak et al. 2020).

## Discussion

Studying the relationship of modern domesticated organisms to ancestral populations of wild relatives has clarified the evolution of dogs (Bergström et al. 2022), silkworms (Tong et al. 2022), and red beans (Chien et al. 2025), helping to elucidate which traits evolved through domestication. Similarly, our results suggest that hallmark features of *A. oryzae* may have begun evolving prior to domestication. Aflatoxin production is reduced in population C (Drott et al. 2020), and the genes in this cluster are sometimes degraded or altogether lost in isolates of population D (Hatmaker et al. 2025); both of these populations share more recent common ancestry with *A. oryzae*, raising the possibility that some softening of mycotoxigenic traits preceded domestication. While the geographic distribution of population D remains unclear, population C is enriched in northern latitudes (Drott et al. 2020; Xie et al. 2026) which aligns with the historical context of *A. oryzae* domestication. Transcriptomic patterns associated with domestication are also evident: *A. oryzae* shows the strongest expression of module 15, enriched in carbohydrate metabolism, with population C following closely. Surprisingly, this module was not significantly enriched when accounting for phylogenetic differentiation, raising questions about the evolution of carbohydrate utilization before domestication. Notably, *A. oryzae* retains the capacity to express many secondary metabolite genes, sometimes mirroring expression patterns seen in other populations.

*A. oryzae* has long been considered safe and non-pathogenic due to the lack of aflatoxin production (Barbesgaard et al. 1992). However, occasional reports of aflatoxin contamination in fermented products associated with strains identified as *A. oryzae* have prompted some researchers to adopt a more cautious stance regarding assumptions of universal atoxigenicity, even though confirmed aflatoxin production by genomically verified *A. oryzae* isolates has not been demonstrated (e.g., Kim et al. 2014). We were therefore surprised to find that *A. oryzae* isolates fall within a clade of *A. flavus* population D enriched in clinical strains (Hatmaker et al. 2025). *A. oryzae* retains pathogenic potential in a Zebrafish model; while a trend towards lower virulence in this group is not definitive from our small sample, this pattern is consistent with past studies (Ford and Friedman 1967; Barbesgaard et al. 1992). Although putative *A. oryzae*-infected samples have been identified from patients (Barbesgaard et al. 1992), these isolates may have been misidentified. Difficulties differentiating *A. oryzae* from *A. flavus* have been lamented in the literature for over a century (Wehmer 1901; Thom and Church 1921). Our results clarify some of the confusion: while *A. oryzae* has previously been reported as monophyletic (Watarai et al. 2019), suggesting a single domestication event, the inclusion of population D isolates resolves *A. oryzae* as polyphyletic. The presence of the *A. flavus* type strain, NRRL1957, in the polyphyletic distribution that includes many industrial isolates argues for a true polyphyly. A similar conclusion was reached based on phylogenomic analysis of *A. flavus* and *A. oryzae* that showed substantial admixture between *A. oryzae* and populations C and D (Pfliegler et al. 2025). While *A. oryzae* could escape from industrial facilities, intermingling with environmental strains, the high clonality of industrial isolates and relative diversity of clinical/environmental isolates does not support this as an explanation for the patterns we observe. Multiple domestications (and/or post-domestication admixture) may better align with historical accounts of ‘spontaneous’ fermentation practices (Allwood et al. 2023). More work is needed to define the boundaries of *A. oryzae* within population D. Although *A. oryzae* shares ancestry with this clinically enriched population, our findings do not support uniformly elevated virulence within this clade. Broader sampling will help in clarifying if virulence may have attenuated before domestication and how population and provenance (i.e. industrial, clinical, and natural sources of isolation) impact virulence.

Recent phylogenomic studies suggest that the evolutionary history of the *A. flavus–oryzae* complex may be substantially more intertwined than previously appreciated, with recombination and admixture potentially playing major roles in shaping domestication-associated lineages (Pfliegler et al. 2025). Our transcriptomic analyses do not resolve these evolutionary relationships, but they do provide a complementary perspective on intraspecific variation. Despite broad sampling across the historically recognized O1 lineage, including both industrial and environmental isolates, we repeatedly recovered transcriptomic signatures that differentiated *A. oryzae* from other populations. For example, module 27 and a hierarchical clustering clade containing the asparasone A, aflatrem, and piperazine backbones were all consistently downregulated in *A. oryzae* regardless of isolation source. Likewise, *A. oryzae* occupied a distinct SM backbone transcriptomic space (Suppl. material 2: fig. S12). Not all patterns were uniform within *A. oryzae*: while ustiloxin B and aflatoxin backbone genes generally showed significantly reduced expression in *A. oryzae*, individual isolates occasionally deviated from these trends. These deviations included isolates from multiple isolation sources, suggesting some biological coherence across the clade. Similar within-population variation was observed in other populations, suggesting that while population-specific transcriptomic signatures are evident, standing variation can still deviate from overarching patterns, consistent with observations that among-population transcriptomic differentiation often coexists with substantial within-population variation (Whitehead and Crawford 2006). Thus, while phylogenetic boundaries within the complex remain under revision, our results suggest that the historic *A. oryzae* lineage does exhibit some coherent transcriptomic signatures across diverse sampling.

Domestication has likely reshaped the ecology of *A. oryzae* by altering its nutrient landscape and promoting growth at unnaturally high densities. These shifts may help explain distinct gene expression patterns observed in domesticated strains: for instance, the overrepresentation of module 15, enriched for carbohydrate metabolism, mirrors findings from earlier studies growing the fungus on rice (Gibbons et al. 2012) and has been interpreted as a signature of *A. oryzae*’s adaptation to food fermentation (Han et al. 2024). However, expression of this module in our study was not significantly differentiated beyond phylogenetic expectation, suggesting that enhanced carbohydrate metabolism may reflect a trait that predates domestication, or that domestication-associated differences in carbohydrate metabolism are more pronounced under fermentation-relevant conditions; indeed, we find that populations can respond differentially to environmental change (discussed in next paragraph). While the expression of many SMs varied between populations (Fig. 3), *A. oryzae* retained the ability to express many SM genes, emphasizing that these genes are not entirely pseudogenized. Although transcriptomic profiles do not always directly correlate with metabolite production (Bergmann et al. 2010; Shostak et al. 2020), many of our findings align with previously reported phenotypes, and transcriptomes provide insight into which pathways are actively engaged. We identify transcriptomic differences between both domesticated and natural lineages, suggesting that large-scale reorganization of gene expression is a general mechanism of microbial adaptation. While fungal evolutionary studies have often avoided focus on transcriptomic divergence — perhaps due to concerns about its heritability — bacterial systems increasingly recognize these traits as both adaptive and sometimes genetically encoded (Ryall et al. 2012). The population-level differences were often consistent across individuals, supporting a model in which heritable regulatory variation contributes to ecological specialization. One striking example is module 22, associated with development and reproduction, which is markedly downregulated in population B — a group with limited recombination and a skewed conidia-to-sclerotium ratio (Drott et al. 2020). These findings position population-specific transcriptomic architectures as a powerful, and underappreciated, lens for studying fungal ecology and evolution.

Population transcriptomic differentiation in *Aspergillus* may confer adaptive flexibility in response to ecological shifts. We examined the effects of spore density — an environmental parameter central to industrial *koji* production and relevant in natural contexts where spore dispersal varies across spatial scales. Prior work has linked spore density to shifts in aflatoxin and sclerotial production via oxylipin signaling (Affeldt et al. 2012). Our results extend this framework by revealing substantial, population-specific transcriptomic rewiring in response to density. While NRRL3357 (2mAO) exhibited strong density-dependent induction of aflatoxin genes, this pattern was not consistent across other isolates. Notably, two SM clusters — asparasone A and aflatrem, both associated with sclerotia — were strongly induced at low densities in populations B and C, but not in *A. oryzae*. While population B is known for prolific sclerotial development, population C typically forms few or no sclerotia (Drott et al. 2020), suggesting that activation of these clusters may not strictly depend on structure formation. This potential decoupling raises questions about other structure-related compounds like xanthones in *Aspergillus nidulans* (Eidam) G. Winter cleistothecia (Liu et al. 2021). Regulatory rewiring across evolutionary timescales has been documented in fungi, including between the non-pathogenic yeast *Saccharomyces cerevisiae* (Desm.) Meyen and the pathogenic yeast *Candida albicans* (C.P. Robin) Berkhout, where shifts in transcription factor-target resulted in shifts of gene expression that the authors speculate may contribute to defining species niches (Dalal et al. 2016). Our results suggest that analogous remodeling of regulatory architecture can occur at the population level within a species complex. Together, these findings underscore the importance of considering transcriptional plasticity not merely as a downstream consequence of development, but as a dynamic, population-specific ecological strategy.

The genetic regulation of secondary metabolism in *Aspergillus* has been widely studied, revealing that BGCs often share co-localized cis-BGC regulatory elements conserved across evolutionary timescales (Woloshuk et al. 1994; Seong et al. 2009; Rösler et al. 2016). Global regulators such as *laeA* orchestrate broad transcriptional control of multiple SMs within a genome (Bok and Keller 2004). However, most of this work has relied on single reference strains, offering limited insight into how regulatory architectures vary within species. Our population-level gene regulatory networks suggest that differences in both secondary and primary metabolism can arise from differentially regulated transcription factors (TFs), rather than changes in core BGC architecture. Notably, we identify evidence for both cis-BGC-acting regulation (e.g., *ustiloxin*) and rare trans-BGC regulation, as seen in a piperazine-associated TF that appears to control multiple unrelated clusters. Although trans-regulatory influences across BGCs are seldom reported, exaptation of regulators has been observed (Bergmann et al. 2010; Shostak et al. 2020; Wang et al. 2021), emphasizing the potential for dynamic interactions between BGC regulators. While further validation is needed to confirm the relationship of specific transcription factors to population-specific transcriptomic patterns, and to clarify how transcriptomic patterns translate into phenotypic differences, these findings suggest that transcriptomic restructuring between populations occurs at a modular scale impacting the expression of one or several metabolites at a time while *laeA* remains stable.

In *A. flavus*, gene regulation, particularly of secondary metabolism, has population-specific responses to ecological context, including spore density. Canonical features of *A. oryzae* domestication like increased carbohydrate metabolism and low aflatoxin production may have begun evolving before domestication; after domestication, *A. oryzae* retains expression of some SMs, at least at the transcript level, and virulence. The distribution of *A. oryzae* isolates suggests multiple domestication events, or admixture. By leveraging population-scale gene expression and inferred regulatory networks, we uncover modular rewiring of population-specific SM pathways, suggesting that cis and trans regulation have both contributed to diversification. Population-specific responses to environmental stimuli could have significant implications for fungal evolution and our understanding of mycotoxigenic threats in agriculture. These results offer a framework for understanding how domestication and population ecology interact to shape fungal phenotypes and raise questions about the evolutionary histories of industrial fungi like *A. oryzae*.

## Additional information

### Artificial Intelligence (AI) use

The authors accept full responsibility for the content of the manuscript, including the disclosure of any use of AI.

No AI tools were used in the preparation of this manuscript.

### Adherence to national and international regulations

All the fungal strains used in this study have been legally obtained, respecting the Convention on Biological Diversity (Rio Convention).

### Author ORCIDs

M.T. Drott https://orcid.org/0000-0001-9715-2200

Y.-W. Wang https://orcid.org/0000-0002-6851-1646

E.A. Hatmaker https://orcid.org/0000-0002-7821-8160

N.M. Mercado-Soto https://orcid.org/0000-0003-3135-9086

J.M. Elmore https://orcid.org/0000-0003-4068-0373

D. Luciano-Rosaro https://orcid.org/0000-0002-9947-4729

J.-H. Yu https://orcid.org/0000-0002-6530-8407

A. Rokas https://orcid.org/0000-0002-7248-6551

J.G. Gibbons https://orcid.org/0000-0003-1282-9345

K. Yang https://orcid.org/0000-0002-9246-101X

N.P. Keller https://orcid.org/0000-0002-4386-9473

### Data availability

All transcriptomic and genomic data generated here have been made available through NCBI project PRJNA1517181. Additionally, all sequences used in the construction of the pangenome presented here and the corresponding summary table output of OrthoFinder are available through Figshare (https://figshare.com/projects/Population-Specific_Transcriptomic_Shifts_Underlie_Secondary_Metabolic_Diversification_in_i_Aspergillus_flavus_i_and_the_Domestication_of_i_Aspergillus_oryzae_i_/280311).

## Supplementary materials

Supplementary material 1 Additional tables Data type xlsx https://binary.pensoft.net/file/1803637This dataset is made available under the Open Database License (http://opendatacommons.org/licenses/odbl/1.0/). The Open Database License (ODbL) is a license agreement intended to allow users to freely share, modify, and use this Dataset while maintaining this same freedom for others, provided that the original source and author(s) are credited. Milton T. Drott, Yen-Wen Wang, E. Anne Hatmaker, Nayanna M. Mercado-Soto, J. Mitch Elmore, Dianiris Luciano-Rosaro, Jae-Hyuk Yu, Antonis Rokas, John G. Gibbons, Anna Huttenlocher, Kunlong Yang, Nancy P. Keller

Supplementary material 2 Supplemental methods and figures Data type docx https://binary.pensoft.net/file/1803638This dataset is made available under the Open Database License (http://opendatacommons.org/licenses/odbl/1.0/). The Open Database License (ODbL) is a license agreement intended to allow users to freely share, modify, and use this Dataset while maintaining this same freedom for others, provided that the original source and author(s) are credited. Milton T. Drott, Yen-Wen Wang, E. Anne Hatmaker, Nayanna M. Mercado-Soto, J. Mitch Elmore, Dianiris Luciano-Rosaro, Jae-Hyuk Yu, Antonis Rokas, John G. Gibbons, Anna Huttenlocher, Kunlong Yang, Nancy P. Keller
